# The Attitude of Great Writers towards Disease

**Published:** 1892-08-20

**Authors:** 


### The Attitude of Great Writers Towards Disease.

### X.—KEATS AND SHELLEY.

To link these names together, in considering the place which disease and pain hold in their poems, is to present a more complete contrast than would naturally be supposed. If one of the two poets could be deemed in the course of his short life to have been brought into closer touch with pain than the other, we should expect that one to be Keats, and we should look to find in his work some evidence of mental struggle, if not a complete revelation of a wounded soul. Nothing could be farther from the mark than such an expectation. The realm of poetry was to Keats a sacred, almost an enchanted, region. He filled it with luscious colouring, languid motion, and ever varying sound and scent. All the strife and dissonance of the world is stilled or heard faintly as a distant echo subdued to harmony. He would have deemed it profanation to introduce the note of personal anguish. Rather, he fled hither from his cares, and found here his purest joy, vexing himself not at all about the causes and cure of the world's sorrow or his own. He is essentially the holiday poet beguiling the tired brain to rest and the restless toiler to enjoy. He stays the passing moment, and crystallises its complex beauty with the magic touch he has ascribed to the sculptor of his Grecian Urn. His diction has all the cloying sweetness of the banquet heaped by Porphyro at Madeline's bedside. His song breathes the same atmosphere of unutterable content and haunting melancholy as that of his own nightingale. And his melancholy is that of which Shelley speaks when he says "the pleasure that is in sorrow is sweeter than the pleasure of pleasure itself." We know that the illusion cannot last. Soon the nightingale's song will cease, the charm will be broken and earth resume its common work-a-day aspect. But it has been good to escape for a time. Roast mutton tastes all the better after that dainty Elysian fare, and the forms of beauty once revealed are ineffaceable.

It is no light debt we owe to the "Cockney poet," that he has shown what visions of purest loveliness may dwell even with those weighed down by uncongenial drudgery and harassed by illness and want.

With Shelley all is different. Birth, means, education, leisure, all the gifts lacking to Keats were his. For him poetry was no refuge from the bitter realities of life; on the contrary, it was a great moral agency for painting them in true colours. Dating even from his Eton days, he was possessed with an overwhelming compassion for the sufferings of others, and a passionate desire to redress their wrongs. Very soon this zeal led him into opposition to established authority, and from that time to the end, the war which he waged against the conventions of society never ceased. To this war his Muse was made subservient, and rarely indeed are his poems free from its influence. Much that seems at first sight unnatural in Shelley's attitude towards society, is explained by his early experiences at home and at the University. He saw himself harshly judged, his motives misunderstood, and his liberty of action hampered; it was but a step from this to the conviction that the origin of pain lies in authority, and that secular law and religion are but so many engines for its infliction. All his long poems are a vindication of this theory. In "The Revolt of Islam" it is the priest and the king together who "have the world at will," while war, pestilence, and famine follow in their train. "The Cenci" is a protest against parental tyranny; "Rosalind and Helen" against the existing marriage laws; while Prometheus Unbound was directed against those crude impressions of a vengeful Deity which had been early forced upon him, and which had contributed more than any other influence to his adoption of the name of Atheist.

All the old existing barriers by which suffering was kept aloof from privileged lives, and which other men held most sacred, were hateful in his eyes; he could see no hope of freedom but in their utter destruction. Not for one moment could he bring himself to be tolerant of intolerance, and the short limits of his life allowed no space for calmer judgment. It is curious how completely this didactic purpose of his art, on which he expended his highest aspirations, and to which he sacrificed all the best of his life, is now regarded as an interruption and source of weakness. Only when at rare moments he let himself rise above the passionate zeal of the politician and reformer was his best work produced. We can never know to what heights he might have attained had time allowed the philosophy so unequally yoked together with his Muse, to reach a full maturity.

In dealing with health and disease the contrast between the two poets is equally complete.

Keats' young men and maidens are all beautiful and healthy; Shelley's are apt to be weighed down with care, hectic of complexion, and short-lived. "Genius and death" contend for mastery, and the victory is to the stronger. One striking peculiarity has been noticed about Keats's young people, and that is their way of fainting. This has been attributed to the poet's own want of bodily vigour, which may have caused him to be easily overpowered by any strong emotion, and to endow his lovers with a similar susceptibility. But fainting, for whatever reason, was much more in fashion in those days than it is now, and was used freely to heighten a situation or extricate a heroine from a difficult position.

Keats excludes disease from his poems, just as he shuns a near approach to thoughts of wrong and sorrow. Shelley will exclude nothing which will enforce a lesson or support a theory, and in his earlier work at least often sacrificed artistic effect to realistic detail—

> Forgetting the great end
> Of poesy; that it should be a friend
> To soothe the cares and lift the thoughts of man.

For any "conclusion of the whole matter," any definite insight into the causes of human suffering, any masterly analysis of its effects, we look in vain in the writings of either poet. And the riddle of their premature death is still unread. Only of this we are sure—

> The splendours of the firmament of time
> May be eclipsed, but are extinguished not;
> Like stars to their appointed height they climb,
> And death is a low mist which cannot blot
> The brightness it may veil.

---

For some time past a New York physician has been making experiments on the hereditary transmission of mutilations, using for the purpose white mice, owing to the rapidity with which they breed. The results have now been published. He bred his small rodents in-and-in for ninety-sixty generations, destroying all the sickly progeny, thus obtaining a larger specimen than the original pair. In order to produce a perfect breed of tailless mice, he selected a pair, placed them in a cage by themselves, and clipped the tails off all the young. When these were old enough to breed he repeated the process with a selected pair, continuing till after obtaining some young in the seventh generation without tails he finally got a breed of tailless mice. A breed of mice with their normal appendages, and doubtless a smirk of satisfaction at their recovery, was again obtained by taking one mouse with a tail and one without, and alternating the sexes in each generation. The result was, no doubt, more satisfactory and interesting to the doctor than to the mice who were his victims.

